# Job Embeddedness Demonstrates Incremental Validity When Predicting Turnover Intentions for Australian University Employees

**DOI:** 10.3389/fpsyg.2016.00582

**Published:** 2016-04-25

**Authors:** Brody Heritage, Jessica M. Gilbert, Lynne D. Roberts

**Affiliations:** ^1^School of Psychology and Exercise Science, Murdoch UniversityMurdoch, WA, Australia; ^2^School of Psychology and Speech Pathology, Curtin UniversityBentley, WA, Australia

**Keywords:** embeddedness, turnover, university, college, validity, measurement

## Abstract

Job embeddedness is a construct that describes the manner in which employees can be enmeshed in their jobs, reducing their turnover intentions. Recent questions regarding the properties of quantitative job embeddedness measures, and their predictive utility, have been raised. Our study compared two competing reflective measures of job embeddedness, examining their convergent, criterion, and incremental validity, as a means of addressing these questions. Cross-sectional quantitative data from 246 Australian university employees (146 academic; 100 professional) was gathered. Our findings indicated that the two compared measures of job embeddedness were convergent when total scale scores were examined. Additionally, job embeddedness was capable of demonstrating criterion and incremental validity, predicting unique variance in turnover intention. However, this finding was not readily apparent with one of the compared job embeddedness measures, which demonstrated comparatively weaker evidence of validity. We discuss the theoretical and applied implications of these findings, noting that job embeddedness has a complementary place among established determinants of turnover intention.

## Introduction

Employee turnover is damaging to organizations. Turnover harms the wellbeing of organizations, as turnover weakens financial, human, and social capital as a consequence of an employee’s knowledge, skills, and social links exiting the workplace (Zhang et al., 2012). Turnover intentions, a key determinant of turnover held by employees (Jiang et al., 2012), is a related concern for organizations as it precipitates the prospect of employee loss. For example, Park and Shaw (2013) recently demonstrated that a one standard deviation increase in turnover intention was related to a 40% loss in organizational productivity and a 26% loss in financial performance. The applied consequences for turnover intentions are demonstrably severe, and have warranted investigation by organizational researchers to account for its determinants.

The process of mapping determinants of turnover and turnover intentions is an established area of inquiry within the body of the literature related to this topic. Price’s (2001) reflection on three decades of research suggested a litany of potential determinants of turnover and turnover intentions (e.g., social support, pay adequacy). However, Mitchell and Lee’s (2001) introduction of job embeddedness as a possible determinant that same year represented an important distinction from previous attempts to map turnover determinants. Mitchell and Lee (2001) described embeddedness as a factor that ‘glued’ employees to their employer, due to the dual influence of both on- and off-the-job factors enmeshing individuals within organizations. Mitchell and Lee’s (2001) embeddedness model represented a new, parsimonious account of predicting turnover and turnover intention. Specifically, the recognition of off-the-job factors that embed an employee within an organization was presented by Mitchell and Lee (2001) as an important area to acknowledge in turnover research. For example, an employee may be enmeshed by the shortness of their commute, or the community links accessed as part of their employment, and these mechanisms may reduce turnover intention. To this end, the model provides a symmetrical consideration of the relevant contributing factors to on- and off-the-job embeddedness, focusing on the influence of perceived Fit, Links, and Sacrifice both within and outside the workplace.

The Fit construct of embeddedness refers to the compatibility between the employee and employing organization (on-the-job), and between the individual and the community (off-the-job). Unlike predictors such as affective organizational commitment or job satisfaction that typically dominate the discussion of turnover intention research, the Fit facet of job embeddedness is non-affective in nature (Lee et al., 2014). It instead focuses on contextual elements to judge congruence, such as whether the organization’s and employee’s values are aligned (Mitchell and Lee, 2001). The Links construct of embeddedness refers to connections (both formal and informal) that occur as part of an individual’s employment, or within their community. Within the workplace, these can refer to relationships with working teams and colleagues, while outside of the workplace links are related to the importance of friends, family, and community groups (Mitchell and Lee, 2001). The Sacrifice construct of embeddedness refers to losses incurred if turnover were to occur, such as severed ties with colleagues or rescinded job perks, or removal from a community due to relocation (Mitchell and Lee, 2001). To summarize, Mitchell and Lee’s (2001) construct of job embeddedness provides a multifaceted account of on- and off-the-job factors that influence why people may want to remain within their organization. However, the measurement approaches to examining embeddedness have attracted calls for further scrutiny on how it is measured (Zhang et al., 2012), as well as general cautions regarding spurious effects in turnover research (Russell, 2013), providing the impetus for further research in these domains when examining the validity of embeddedness.

The measurement of job embeddedness is an area that warrants further investigation, with three competing measures of job embeddedness currently available: Mitchell and Lee’s (2001) six subscale job embeddedness measure, Crossley et al. (2007, 2011) global job embeddedness measure, and Clinton et al.’s (2012) two subscale measure. As the response methods, scope, and parsimony of each measure may influence the criterion validity of these measures, further scrutiny of the measurement methods of job embeddedness is warranted.

Stemming from Mitchell and Lee’s (2001) seminal article, their six subscale job embeddedness measure comprised multiple indicators for both on- and off-the-job facets of fit, links, and sacrifice. As such the content validity is high. However, the measure is limited to collecting formative data for its Links subscales, which means it is not suitable for analysis using procedures such as structural equation modeling which rely on latent factors driving measured indicators (Zhang et al., 2012). Our study, therefore, focuses on two competing attitudinally reflective measures of job embeddedness, Crossley et al.’s (2007) measure and Clinton et al.’s (2012) measure.

A reflective measurement approach to job embeddedness was articulated by Crossley et al. (2007, 2011) global job embeddedness measure, which is a shortened seven item version of Mitchell and Lee’s (2001) 40 item questionnaire. Zhang et al.’s (2012) review of the measurement and conceptualisation of job embeddedness reported that respondents experienced difficulties in distinguishing between the on- and off-the-job factors reflected in this measure. While Crossley et al.’s (2011) clarification article noted that individuals were free to consider on- and off-the-job facets simultaneously when considering responses to its items, it is impossible to disentangle the on- and off-the-job facets during analysis due to the lack of distinction between the two during measurement. This is an important limitation, given that Jiang et al.’s (2012) later meta-analysis noted differential relationships between on- and off-the-job indicators with voluntary turnover and turnover intentions.

A later version of a reflective job embeddedness measure by Clinton et al.’s (2012) two subscale measure included both on- and off-the-job subscales for fit, links, and sacrifice. This measure has demonstrated acceptable psychometric properties in a military sample and information technology sample. Currently, Clinton et al.’s (2012) measure has received no use outside of its preliminary article, raising questions of its validity outside of the original samples. In summary, the current reflective measures of job embeddedness have recognized limitations, and the convergent validity of these measures has yet to be assessed. These issues, therefore, warrant further examination in a research context.

Furthermore, authors such as Russell (2013) have cautioned against future research on turnover that bypasses determinants with demonstrated validity in previous research contexts. More pointedly, Russell emphasizes the importance of evidencing the incremental variance associated with the introduction of a new predictor of voluntary turnover intentions, as a means of avoiding explanatory plateauing. Consequently, the construct of embeddedness has predictive utility only if it explains incremental variance in voluntary turnover beyond that of the established predictors derived from existing research. There is therefore a clear need for research that examines the predictive utility of the competing job embeddedness measures.

The current research examines the convergent validity and predictive utility (criterion and incremental validity) of the two reflective embeddedness measures (Crossley et al., 2011; Clinton et al., 2012) in the Australian higher education sector. Embeddedness has not previously been studied within this sector, adding a component of contextual novelty to our investigation of the predictive utility of reflective job embeddedness measures. Previous research has identified a range of key determinants of turnover intention among university employees, affording an investigation of the incremental validity of embeddedness beyond these determinants. Pop-Vasileva et al.’s (2011) recent study reported that approximately a quarter of Australian academic staff surveyed were actively seeking employment options at other universities. A key determinant of turnover intention in university employees is job-related stress, as evidenced by numerous studies of this sector (Taris et al., 2001; Winefield and Jarrett, 2001; Winefield et al., 2003, 2008; Conklin and Desselle, 2007; Catano et al., 2010; Mark and Smith, 2011; Pop-Vasileva et al., 2011). Ryan et al. (2012) demonstrated that a one standard deviation increase in stress doubled intention to turnover within a university employee cohort. While the previous research was not conducted solely within Australia and varied in its inclusion of academic and professional university employees, the consistency of findings regarding stress’ importance as a determinant of turnover intentions is notable. A related concept to stress, burnout, has similarly demonstrated relevance to the turnover intentions of university staff (e.g., Taris et al., 2001). Stress and burnout are therefore established predictors to be controlled for in a test of embeddedness’ incremental validity. Diminished job satisfaction is another demonstrated predictor of turnover and turnover intention (Jiang et al., 2012), and this construct has seen frequent attention within the context of research involving university employees (e.g., Winefield and Jarrett, 2001; Winefield et al., 2003; Seifert and Umbach, 2008; Catano et al., 2010; Bozeman and Gaughan, 2011). Job satisfaction is, therefore, another key variable to control for in a test of embeddedness’ incremental validity. Furthermore, the perceived availability of other jobs has been recognized as a key predictor of turnover intentions in a recent meta-analysis for a general employee sample (Jiang et al., 2012), which is likely to be transferable to the context of university employees. University employees, particularly academic staff, are typically placed against a very competitive field of potential candidates when attempting to find work elsewhere (May et al., 2013). Therefore, this facet is of importance in establishing the incremental validity of embeddedness indicators. In summary, stress, burnout, job satisfaction, and the perceived availability of other jobs are established determinants of turnover intention within the context of university employees. Statistically controlling for each of these constructs will provide the basis for testing the incremental validity of job embeddedness as a predictor of turnover intentions.

This research will also provide the opportunity to scrutinize the convergent and criterion validity of two competing reflective measures of embeddedness: Crossley et al.’s (2011) measure and Clinton et al.’s (2012) measure. The convergent validity of these measures, when compared, has theoretical importance. Basic correlation of the measures’ scores should be high, as the measures are purported to tap into the same underlying constructs (on- and off-the-job embeddedness). If the measures demonstrate convergence, parsimony would suggest that Crossley et al.’s (2011) substantially shorter scale would be preferable. Results that deviate from this expected outcome would suggest that the measures are not providing equivalent information, despite the instrument design goals of the authors. This would raise theoretically important questions as to what is being measured by these instruments. Regarding the criterion validity of the measures, this similarly has theoretical merits to warrant its investigation. Either measure of job embeddedness should demonstrate significant bivariate correlations with the outcome measure of turnover intention. Clinton et al.’s (2012) measure affords the opportunity to scrutinize whether it is capable of demonstrating criterion validity at the subscale level (on- and off-the-job embeddedness). If Clinton et al.’s (2012) measure demonstrates criterion validity, then this would support its value as a measurement instrument outside of its current lone use in the literature in its validation paper.

To address the issues of measure convergence and predictive utility, we therefore propose the following hypotheses. Firstly, an examination of the degree of relatedness between the two measures of embeddedness as an indicator of convergent validity will be conducted.

H1: Crossley et al.’s (2011) and Clinton et al.’s (2012) measures of job embeddedness will be strongly, and positively, correlated.

Furthermore, scrutiny of the bivariate correlations between the two measures of embeddedness and turnover intentions will be conducted.

H2: Crossley et al.’s (2011) and Clinton et al.’s (2012) measures of job embeddedness will be negatively correlated with turnover intentions.

As discussed prior, several key predictors of turnover intention in previous samples of university employees have been noted: stress, burnout, the perceived availability of other jobs, and job satisfaction. Therefore, these variables will be controlled for during the testing of the forthcoming incremental validity hypotheses. While disadvantages exist for both reflective measures of job embeddedness, both embeddedness measures should be capable of accounting for unique variance if incremental validity is supportable in a university context. In testing the two measures of job embeddedness in predicting turnover intention in the Australian tertiary education system, we propose the following hypotheses:

H3: Crossley et al.’s (2011) global job embeddedness measure will predict significant variance in turnover intention for university employees, after accounting for known predictors of turnover intention.
H4: Clinton et al.’s (2012) dual subscale job embeddedness measure will predict significant variance in turnover intention for university employees, after accounting for known predictors of turnover intention.

## Materials and Methods

### Participants

A convenience sample of 246 Australian university employees from 20 universities voluntarily participated in the research. As the number of participants per university was untenable for an intra-class correlation coefficient to be calculated (i.e., many university groups *n* < 10), nested effects could not be estimated for this sample. The sample included 78 male (*M*_age_ = 42.67 years, *SD*_age_ = 10.70) and 167 female (*M*_age_ = 40.82 years, *SD*_age_ = 10.55) participants, with one participant not listing their gender. Most participants were academic staff (59.3%), with the remainder identifying as professional staff. The sample was mostly employed on a full-time basis (71.5%), with 17.1% of participants employed part-time, 11.0% employed as sessional staff, and one staff member not listing their mode of employment. The majority of participants were employed on an on-going basis (56.9%), with the remainder being on fixed-term contracts.

### Measures

#### Job Stress

Job stress was measured using Rizzo et al.’s (1970) Role Conflict and Role Ambiguity scales. Participants were asked how true each of the 14 statements presented in the questionnaires are of their job, and responded on a seven-point Likert-style scale ranging from 1 “Very True” to 7 “Very False.” An example item from the role ambiguity subscale was “I know exactly what is expected of me.” An example item from the role conflict subscale was “I work with two or more groups who operate quite differently.” The measure has previously demonstrated acceptable psychometric properties: α = 0.89 for both scales, and evidence of construct validity and criterion validity (Fisher and Gitelson, 1983; Jackson and Schuler, 1985; Tubre and Collins, 2000).

#### Burnout

Burnout was measured using the Burnout Measure Short Version (BMS; Malach-Pines, 2005). The BMS consisted of 10 items that measure participants’ reported emotional, mental, and physical exhaustion. Participants were asked to consider how the items relate to their feelings about work, and responded on a 1 (“never”) to 7 (“always”) Likert-style scale for each item. An example item was “Difficulties sleeping” (Malach-Pines, 2005, p. 88). The author reported acceptable reliability across multiple samples, α = 0.85 to 92, a unidimensional burnout factor structure, and criterion validity with other expected variables such as work satisfaction (Malach-Pines, 2005).

#### Job Satisfaction

Job satisfaction was measured using the abridged version of the Job in General scale [aJIG] (S. Ironson et al., 1989; Russell et al., 2004). The scale contained eight items which provided the participant with an affective judgment of the job at a global level. The aJIG has demonstrated excellent internal consistency and psychometric properties during its validation (Russell et al., 2004).

#### Job Embeddedness

Two reflective scales measured job embeddedness: the Global Job Embeddedness Scale (GJES; Crossley et al., 2007, 2011), and the job embeddedness measure of Clinton et al. (2012). Crossley et al.’s (2007, 2011) GJES consisted of seven statements designed to tap into both the on- and off-the-job aspects of job embeddedness, to which participants responded using a five-point Likert-style scale, ranging from 1 (“strongly disagree”) to 5 (“strongly agree”). An example item from the GJES was “I feel tied to this organization” (Crossley et al., 2011, p. 1316). Evidence of adequate internal consistency (α = 0.89) and validity has been presented by Crossley et al. (2007).

Clinton et al.’s (2012) measure consisted of 12 items (six on-the-job, six off-the-job) measuring job embeddedness. Clinton et al.’s (2012) measure used the same five-point Likert-style scale as that of Crossley et al. (2007) and has demonstrated construct validity and reliability in their validation of the measure in a sample of military and IT employees. An example on-the-job subscale item from Clinton et al.’s (2012, p. 114) scale was “Overall, I have strong ties with people throughout [the organization].” An example off-the-job subscale item is “Even if I decide to leave [the organization] I would still live in the area where I am based at the moment.”

#### Job Alternatives

Consistent with the choice of measure for job alternatives by Crossley et al. (2007), we used three items from Steel and Griffeth’s (1989) summary of job alternatives measurement items. An example item was “I know of several job alternatives that I could apply for” (Crossley et al., 2007, p. 1035). Scale reliability was acceptable (α = 0.69), and predictive criterion validity was established with regards to outcomes such as turnover intention (Crossley et al., 2007).

#### Voluntary Turnover Intentions

Turnover intention was measured using a three-item scale (Jaros, 1997). Participants responded via a five-point Likert-style scale, with scale endpoints tied to statements that asked how often (1 = very rarely, 5 = very often) they considered leaving their employer, or how likely (1 = very unlikely, 5^1^ = very likely) it is that they would leave their job in the near future. Adequate scale reliability has been demonstrated, with coefficient α that ranged from 0.81 to 0.85 (Meyer et al., 1993; Jaros, 1997).

#### Demographic Items

Participants were asked to provide their age (in years), gender, role within the university (academic or professional staff), and mode of employment (full-time, part-time, sessional; contract, on-going).

### Design

The study used a cross-sectional, correlation design. To minimize the prospect of common method variance serving as a source of response bias, several approaches were taken during the design of the questionnaire. To lower the probability of socially desirable responding (a contributor to common method bias), we acknowledged prominently in the participant information precluding the study that all results would be anonymously collected, with individual responses being unidentifiable (Chang et al., 2010). We controlled for acquiescence-biased responding through careful selection of measures which varied in their scale endpoints (Chang et al., 2010). To this end, the two of our measures which did share scale endpoints were the two job embeddedness measures, and these measures were not included in the same regression models tested later in the article. Furthermore, our measures were selected based on conciseness (e.g., the Abridged Job In General scale) to reduce the prospect of participant fatigue influencing patterned responding. As participant responses on the turnover intention scale may have been influenced by the items from other measures, prompting the participant to consider their intentions in light of their responses to measures of burnout and other similar variables, we forced the turnover intention items to be the first items responded to by the participant. Otherwise, the ordering of measures, and the item ordering within these measures, varied randomly for each participant to further diminish the likelihood of patterned responding (Chang et al., 2010). The bivariate correlations presented later in the article (see **Table 1**) are additionally indicative of the lack of relatedness based on common methods (Spector, 2006). Approximately 25% of our correlations were non-significant, despite the analysis being sufficiently powered to detect small-to-moderate effect sizes. While, we cannot rule out the prospect of common method bias serving as a source of error within the current study, our design and descriptive statistics would suggest that this issue has been addressed sufficiently within the limitations inherent in cross-sectional research.

**Table 1:** Scale means and standard deviations per demographic indicator (*N* = 246).

	Gender^a^		University role		Mode of employment^a^		
	Males	Females	Academic	Professional	Full-time	Part-time	Sessional
Turnover intention	9.40 (3.56)	8.65 (3.83)	8.73 (3.64)	9.12 (3.93)	9.09 (3.81)	8.35 (3.58)	8.37 (3.66)
Role ambiguity	27.60 (7.98)	27.75 (7.98)	26.37 (7.63)	29.58 (8.08)	27.85 (7.88)	28.26 (7.45)	26.07 (9.11)
Role conflict	36.94 (10.36)	34.45 (11.87)	37.33 (10.65)	32.31 (11.96)	36.45 (11.58)	32.05 (11.08)	32.36 (9.98)
Job satisfaction	15.29 (8.10)	16.21 (7.30)	16.11 (7.46)	15.51 (7.77)	15.79 (7.77)	15.76 (6.72)	16.74 (7.85)
Burnout	35.58 (12.83)	37.50 (13.31)	37.61 (12.45)	36.03 (14.23)	38.45 (13.43)	34.43 (11.29)	30.93 (12.59)
Global JE	17.25 (6.29)	18.48 (6.31)	18.14 (6.27)	17.91 (6.46)	17.91 (6.28)	18.45 (6.08)	18.46 (7.29)
JE_on_	18.63 (4.88)	19.51 (4.29)	19.60 (4.48)	18.59 (4.54)	19.09 (4.63)	19.71 (3.16)	19.07 (5.64)
JE_off_	22.63 (4.73)	23.21 (4.83)	23.18 (5.10)	22.64 (4.54)	22.66 (5.08)	23.64 (4.44)	23.85 (4.20)
JE_total_	41.26 (7.67)	42.72 (7.04)	42.78 (7.54)	41.23 (7.15)	41.75 (7.58)	43.36 (6.03)	42.93 (8.31)
*N*	78	167	146	100	176	42	27

*^a^One participant did not provide data for this demographic item. Global JE = global job embeddedness per Crossley et al.’s (2011) scale. JE_on_ = on-the-job embeddedness per Clinton et al.’s (2012) subscale. JE_off_ = off-the-job embeddedness per Clinton et al.’s (2012) subscale. JE_total_ = sum of JE_on_ and JE_off_ per Clinton et al.’s (2012) subscales.*

### Procedure

Following Human Research Ethics Committee approval from Curtin University (PSYCH SP 2014-10), we advertised the study in November 2014 to university employees via email, social media (Twitter, Facebook, LinkedIn), and flyers at conferences. We used an electronic questionnaire hosted on Qualtrics.com to collect participant data, including a separate questionnaire and database to record participant prize draw entries (one $100 Amazon.com gift voucher). We closed the study in mid-December to coincide with the holiday shutdown at Australian universities. Data was downloaded by the researchers as an SPSS (IBM Corporation, Armonk, NY, USA) data file before conducting data analysis.

## Results

### Descriptive and Correlational Data for the Sampled University Employees

Little’s test for data missingness indicated that data from the items was missing completely at random, χ^2^ (890) = 793.69, *p* = 0.991, and missing data was imputed using expectation maximization before proceeding. **Table 1** presents the means and standard deviations of each measure by employee demographic characteristics. Univariate normality and multivariate assumption testing indicated no concerns outside of the moderate negative skew for the job satisfaction scale score, Clinton et al.’s (2012) off-the-job subscale, and the total scale score. Algebraic transformation was conducted to bring the data closer to univariate normality before analysis. **Table 2** presents the correlations, means, standard deviations and alpha reliabilities of each scale measure for the whole sample.

**Table 2:** Bivariate correlations, scale reliabilities, means, and standard deviations of scales for university staff (*N* = 246).

	TI	RA	RC	JS	Burnout	JA	Global JE	JE_on_	JE_off_	JE_total_
TI	0.872	-0.258***	0.369***	-0.597***	0.536***	0.290***	-0.545***	-0.407***	-0.125	-0.325***
RA		0.876	-0.451***	0.484***	-0.467***	0.000	0.233***	0.267***	0.094	0.202**
RC			0.885	-0.502***	0.573***	0.120	-0.191**	-0.198**	0.067	-0.069
JS				0.897	-0.696***	-0.136*	0.469***	0.576***	0.099	0.413***
Burnout					0.931	0.062	-0.287***	-0.390***	-0.040	-0.253***
JA						0.784	-0.330***	-0.131*	0.086	-0.029
Global JE							0.876	0.549***	0.300***	0.530***
JE_on_								0.797	0.242***	0.770***
JE_off_									0.843	0.790***
JE_total_										0.814
*M*	8.89	27.67	35.29	3.28	36.97	8.80	18.05	19.19	3.32	3.51
*SD*	3.74	7.96	11.45	1.31	13.20	2.94	6.33	4.52	0.92	0.92

*Scale reliabilities (α) are represented along the diagonal. TI = turnover intention; RA = role ambiguity; RC = role conflict; JS = job satisfaction; JA = availability of job alternatives. Global JE = Crossley et al.’s (2011) global job embeddedness total score; JE_on_ = Clinton et al.’s (2012) on-the-job embeddedness subscale; JE_off_ = Clinton et al.’s (2012) off-the-job embeddedness subscale; JE_total_ = Clinton et al.’s (2012) job embeddedness total score. M and SD for aJIG, JE_off_, and JE_total_ are reflective of data post-transformation via square root to improve univariate normality. ^∗^p < 0.05; ^∗∗^p < 0.01; ^∗∗∗^p < 0.001.*

In support of Hypothesis 1, which predicted significant, positive overlap between the measures of Crossley et al. (2011) and Clinton et al. (2012), the magnitude of the correlation between the scales’ total scores was indicative of a large effect size per Cohen’s (1992) conventions, *r* = 0.530, *p* < 0.001. The correlations between Crossley et al.’s (2011) measure and the subscales of Clinton et al.’s (2012) were moderate in effect size. Hypothesis 2 was generally supported by the bivariate correlations that were conducted. Crossley et al.’s (2011) global job embeddedness measure was significantly and negatively correlated with turnover intentions, *r*(244) = -0.55, *p* < 0.001, and was reflective of a strong effect. The measure of Clinton et al. (2012) varied in its demonstration of criterion validity. When considered as a total scale score (combining the on- and off-the-job subscales), the measure demonstrated criterion validity in the expected direction, *r*(244) = -0.33, *p* < 0.001 (a moderate-to-large effect). When considered as subscales, the measure demonstrated partial support for criterion validity. The on-the-job embeddedness subscale was significant and demonstrated a moderate-to-large effect size, *r*(244) = -0.41, *p* < 0.001. The off-the-job embeddedness subscale of Clinton et al. (2012) did not, *r*(244) = 0.13, *p* = 0.05, and reflected a small effect size. In summary, criterion validity for the embeddedness measures was generally supported by the bivariate correlation results, although the off-the-job embeddedness subscale did not provide support for H2.

To examine whether there were significant differences on the demographic variables of gender, mode of employment, and university role (see **Table 1**) prior to the forthcoming regression analyses, *t*-tests and between-groups ANOVAs were conducted using Bonferroni-corrected α to assess significance. While almost all of the comparisons were non-significant, we identified significant (*p* < 0.005) differences between Academic and Professional staff on the stress measures of role conflict and role ambiguity. These findings suggested that treating the participants as part of a homogenous sample for the forthcoming regressions may not be appropriate. Consequently, we conducted the forthcoming regression analyses separately for Academic and Professional university staff participants.

As part of the models created to test the incremental validity of the embeddedness measures, the established determinants of turnover intention were loaded in the first step of hierarchical multiple regression analyses. For professional staff, the set of established determinants collectively accounted for a significant proportion of the variance in turnover intentions, *F*(5,94) = 17.38, *p* < 0.001, *R*^2^ = 0.48, *f*^2^ = 0.92 (a large effect). **Table 3** demonstrates the coefficients for each determinant, with all entered variables besides the two stress scales accounting for significant variance. An equivalent regression analysis was conducted for the academic university staff sample (see **Table 4**). Collectively the entered variables accounted for a significant proportion of variance in turnover intentions, *F*(5,140) = 20.74, *p* < 0.001, *R*^2^ = 0.43, *f*^2^ = 0.75 (a large effect).

**Table 3:** Predictor coefficients, significance, and confidence intervals of the models testing job embeddedness’ incremental validity for professional university staff (*N* = 100).

	*B*	*SE*	95% *LCI*	95% *UCI*	β	*sr*^2^
Block 1						
Constant	4.864	2.536				
Role ambiguity	0.089	0.048	-0.005	0.183	0.184	0.019
Role conflict	-0.012	0.030	-0.071	0.047	-0.037	0.001
Job satisfaction	-1.262	0.359	-1.975	-0.550	-0.442***	0.068
Burnout	0.083	0.033	0.017	0.148	0.302*	0.035
Job alternatives	0.345	0.112	0.122	0.568	0.246**	0.052
Block 2a (Crossley)						
Constant	7.769	2.324				
Role ambiguity	0.102	0.042	0.018	0.186	0.212*	0.026
Role conflict	0.003	0.027	-0.050	0.056	0.010	0.000
Job satisfaction	-0.777	0.333	-1.437	-0.116	-0.272*	0.024
Burnout	0.077	0.029	0.019	0.136	0.282*	0.030
Job alternatives	0.247	0.102	0.045	0.449	0.176*	0.026
Crossley JE	-0.239	0.047	-0.332	-0.146	-0.395***	0.114
Block 2b (Clinton dual scales)						
Constant	7.118	2.668				
Role ambiguity	0.129	0.049	0.032	0.226	0.266**	0.037
Role conflict	-0.001	0.029	-0.059	0.058	-0.003	0.000
Job satisfaction	-1.044	0.374	-1.786	-0.302	-0.366**	0.041
Burnout	0.083	0.032	0.018	0.147	0.302*	0.034
Job alternatives	0.343	0.111	0.122	0.564	0.244**	0.050
Clinton JE _on_	-0.124	0.084	-0.291	0.043	-0.144	0.011
Clinton JE _off_	-0.669	0.351	-1.366	0.027	-0.151	0.019
Block 2c (Clinton total scale)						
Constant	8.038	2.752				
Role ambiguity	0.127	0.048	0.030	0.223	0.262*	0.036
Role conflict	-0.003	0.029	-0.061	0.055	-0.010	0.000
Job satisfaction	-1.070	0.356	-1.777	-0.362	-0.375**	0.047
Burnout	0.081	0.032	0.017	0.144	0.293*	0.033
Job alternatives	0.340	0.109	0.123	0.557	0.242**	0.051
Clinton JE_total_	-0.123	0.048	-0.217	-0.028	-0.225*	0.035

*LCI = lower-bound confidence interval; UCI = upper-bound confidence interval. sr^2^ = squared semi-partial correlation coefficient. Job alternatives = availability of job alternatives. Crossley JE = Crossley et al.’s (2011) global measure of job embeddedness scores; Clinton JE_on_ = Clinton et al.’s (2012) on-the-job embeddedness subscale; Clinton JE_off_ = Clinton et al.’s (2012) off-the-job embeddedness subscale; Clinton JE_total_ = total score for Clinton et al.’s (2012) on- and off-the-job embeddedness subscales. For job satisfaction, Clinton JE_off_, and Clinton JE_total_, the B, SE, and LCI/UCI coefficients are based on the algebraically transformed version of the variables to address univariate normality. ^∗^p < 0.05; ^∗∗^p < 0.01; ^∗∗∗^p < 0.001.*

**Table 4:** Predictor coefficients, significance, and confidence intervals of the models testing job embeddedness incremental validity for academic university staff (*N* = 146).

	*B*	*SE*	95% *LCI*	95% *UCI*	β	*sr*^2^
Block 1						
Constant	8.012	2.321				
Role ambiguity	-0.011	0.035	-0.080	0.059	-0.022	0.000
Role conflict	0.026	0.029	-0.032	0.084	0.076	0.003
Job satisfaction	-1.180	0.251	-1.676	-0.684	-0.416***	0.091
Burnout	0.055	0.028	0.001	0.110	0.190*	0.016
Job alternatives	0.214	0.078	0.059	0.369	0.179**	0.031
Block 2a (Crossley)						
Constant	10.013	2.312				
Role ambiguity	-0.007	0.034	-0.074	0.060	-0.014	0.000
Role conflict	0.030	0.028	-0.026	0.085	0.087	0.004
Job satisfaction	-0.829	0.263	-1.348	-0.310	-0.292**	0.038
Burnout	0.061	0.027	0.008	0.114	0.209*	0.020
Job alternatives	0.110	0.082	-0.051	0.271	0.092	0.007
Crossley JE	-0.150	0.044	-0.236	-0.063	-0.259**	0.045
Block 2b (Clinton dual scales)						
Constant	9.070	2.466				
Role ambiguity	-0.012	0.035	-0.081	0.057	-0.025	0.000
Role conflict	0.033	0.030	-0.025	0.091	0.097	0.005
Job satisfaction	-1.114	0.285	-1.677	-0.552	-0.393***	0.063
Burnout	0.053	0.028	-0.002	0.108	0.183	0.015
Job alternatives	0.220	0.079	0.065	0.375	0.184**	0.032
Clinton JE _on_	-0.011	0.064	-0.137	0.115	-0.014	0.000
Clinton JE _off_	-0.375	0.256	-0.882	0.132	-0.098	0.009
Block 2c (Clinton total scale)						
Constant	9.312	2.519				
Role ambiguity	-0.012	0.035	-0.081	0.057	-0.025	0.000
Role conflict	0.033	0.030	-0.026	0.091	0.096	0.005
Job satisfaction	-1.055	0.268	-1.584	-0.525	-0.372***	0.063
Burnout	0.054	0.028	0.000	0.109	0.186	0.016
Job alternatives	0.215	0.078	0.060	0.370	0.180**	0.031
Clinton JE _total_	-0.044	0.034	-0.111	0.023	-0.092	0.007

*LCI = lower-bound confidence interval; UCI = upper-bound confidence interval. sr^2^ = squared semi-partial correlation coefficient. Job alternatives = availability of job alternatives; Crossley JE = Crossley et al.’s (2011) global measure of job embeddedness scores; Clinton JE_on_ = Clinton et al.’s (2012) on-the-job embeddedness subscale; Clinton JE_off_ = Clinton et al.’s (2012) off-the-job embeddedness subscale; Clinton JE_total_ = total score for Clinton et al.’s (2012) on- and off-the-job embeddedness subscales. For job satisfaction, Clinton JE_off_, and Clinton JE_total_, the B, SE, and LCI/UCI coefficients are based on the algebraically transformed version of the variables to address univariate normality. ^∗^p < 0.05; ^∗∗^p < 0.01; ^∗∗∗^p < 0.001.*

### Incremental Validity of Crossley et al.’s (2011) Measure

To examine the incremental validity of Crossley et al.’s (2011) embeddedness measure, the previous regression models were explored further via the introduction of a second hierarchical step in the analyses. Scores on Crossley et al.’s (2011) measure were entered in this step of the regression model. In the professional staff sample, the introduction of this step accounted for a significant increase in variance, Δ*F* (1,93) = 26.10, *p* < 0.001, Δ*R*^2^ = 0.11, indicative of a small-to-medium unique effect size, *f*^2^ = 0.12. The negative valence of global job embeddedness was in a theoretically consistent direction; as embeddedness increased, the intention to turnover decreased. The introduction of the embeddedness scores in the second step rendered the role ambiguity subscale of the stress measure significant (*p* = 0.017), suggesting a suppressor effect (see **Table 3**). When an equivalent model test was conducted with the academic staff subsample, Crossley et al.’s (2011) measure again predicted significant unique variance, Δ*F* (1,139) = 11.72, *p* = 0.001, Δ*R*^2^ = 0.11, *f*^2^ = 0.05 (a small-to-moderate effect). The introduction of the embeddedness determinant in the academic subsample rendered perceived job alternatives non-significant (*p* = 0.179). Coefficients for each block of predictors are presented in **Table 4**. In summary, support for the incremental validity of Crossley et al.’s (2011) embeddedness measure was reflected in these analyses, supporting H3.

### Incremental Validity of Clinton et al.’s (2012) Measure

The incremental validity of the separate on- and off-the-job embeddedness subscales of Clinton et al.’s (2012) measure were tested via hierarchical multiple regression analysis. Building off the models for professional and academic university staff described in Section “Descriptive and Correlational Data for the Sampled University Employees,” we added a further step to the model that entered the on- and off-the-job embeddedness determinants. For the professional staff subsample, the two determinants added in step two collectively contributed significant variance to the model, Δ*F*(2,92) = 3.55, *p* = 0.033, Δ*R*^2^ = 0.04, a small change in effect size, Δ*f*^2^ = 0.04. While this step significantly accounted for additional variance collectively, the individual predictors did not significantly account for unique variance when considered individually (*p* > 0.05, see **Table 3**). To examine whether the total score of both scales (on- and off-the-job) may account for significant variance in turnover intentions in light of the non-significant determinant-level findings, the total score of Clinton et al.’s (2012) measure was used in an alternative model for the second block of the regression analysis. This version of the model indicated significant variance in turnover intentions accounted for by the total score version of Clinton et al.’s (2012) measure, Δ*F* (1,93) = 6.67, *p* = 0.011, Δ*R*^2^ = 0.04, Δ*f*^2^ = 0.04, a small effect (see **Table 3**). To examine whether these relationships were consistent in the academic staff subsample, equivalent model tests were conducted. Entering on- and off-the-job scales in the second step of analysis did not contribute a significant proportion of explained variance to the model, Δ*F* (1,138) = 1.20, *p* = 0.303, Δ*R*^2^ = 0.01, Δ*f*^2^ = 0.01, a marginal effect. Entering the total score of both scales in the second step contributed similarly non-significant and marginal variance, Δ*F* (1,139) = 1.72, *p* = 0.192, Δ*R*^2^ = 0.01, Δ*f*^2^ = 0.01. To summarize, support for Clinton et al.’s (2012) on- and off-the-job embeddedness measure was weakly provided by the current analyses. Small effects were found within the professional staff subsample, but only for the total score of the scale, and not its subscales. The academic staff subsample results indicated no meaningful effect. Therefore support for Hypothesis 4, which proposed that Clinton et al.’s (2012) measure would demonstrate incremental validity, was weak within the professional staff subsample, and absent within the academic staff subsample.

## Discussion

The intent of this study was to evaluate the incremental, convergent, and criterion validity of job embeddedness (Mitchell and Lee, 2001), an emerging predictor of turnover and turnover intentions in work settings, within a sample of Australian university staff. Crossley et al.’s (2007, 2011) global job embeddedness measure, and Clinton et al.’s (2012) dual scale job embeddedness measure, were examined in this study. There was a moderate degree of convergence between total scores of the measures, although this convergence was weaker when comparing the convergence between Clinton et al.’s (2012) embeddedness subscales and Crossley et al.’s (2007, 2011) global scale. This finding supported our first hypothesis. Partial support for the second hypothesis was also provided by our results, as criterion validity was generally demonstrated by the bivariate correlation results with the exception of the off-the-job subscale of Clinton et al.’s (2012) measure. Our incremental validity testing results indicated that Crossley et al.’s (2007, 2011) global job embeddedness measure accounted for a significant proportion of unique variance in turnover intentions as anticipated, consistent with previous findings in other employment sectors (Jiang et al., 2012; Zhang et al., 2012). This evidence supported our third hypothesis, although it is worth noting that the effect sizes of these relationships were only small-to-moderate. A strength of this finding, however, is that the professional and academic staff subsamples demonstrated a consistent pattern of results. In contrast, the on- and off-the-job subscales of Clinton et al.’s (2012) measure did not account for unique variance in turnover intentions. This finding was contrary to the findings reported by Clinton et al. (2012). When examined as a total score, the measure was able to account for significant unique variance in turnover intentions for professional university staff, although not in the case of academic university staff. Furthermore, there was a notable disparity in effect size compared to the analyses that involved Crossley et al.’s (2011) measure. Approximately 1–4% of the unique variance in turnover was explained by Clinton et al.’s (2012) measure, in comparison to the 7% of unique variance explained by Crossley et al.’s (2011) measure. These findings provided very limited support for our fourth hypothesis, which suggested that Clinton et al.’s (2012) measure would demonstrate incremental validity. When considered in combination, our findings support the inclusion of the construct of job embeddedness when exploring the turnover intentions of university employees. Job embeddedness accounts for unique variance in turnover intention, after accounting for established determinants, although the choice of measure appears to be the caveat to this finding.

### Comparisons between Embeddedness Measures

Clinton et al.’s (2012) measure of job embeddedness was selected for use in this research as it provided the ability to disambiguate the on- and off-the-job elements ‘gluing’ the employee to their job. However, the poor incremental validity of Clinton et al.’s (2012) measure in predicting turnover intentions means the further use of this measure is difficult to support, especially within the context of the higher education sector. Clinton et al. (2012) noted in their validation article that on-the-job embeddedness was a significant predictor of turnover intention, beyond that of organizational commitment, employability, job satisfaction, and demographic variables. This finding was not mirrored by Clinton et al. (2012) for off-the-job embeddedness though, which lends support to our findings of it being a marginal determinant of turnover intentions. Furthermore, the absence of effect size indicators in Clinton et al.’s (2012) study, coupled with the substantial sample size (*N* = 21682), raises the question as to whether the significant on-the-job embeddedness result was an artifact of sample size (type I error). Due to the applied nature of turnover research, the limited support for the incremental validity of Clinton et al.’s (2012) measure of job embeddedness tempers our support for this approach to measuring job embeddedness, especially within a university employee sample.

The strength of the relationships between Crossley et al.’s (2007, 2011) global measure and Clinton et al.’s (2012) measure additionally warrants discussion. **Table 2** demonstrated that Crossley et al.’s (2007, 2011) and Clinton et al.’s (2012) measures’ subscales and total score varied in correlational strength between *r* = 0.30 and 0.55, reflective of a moderate to large effect. While a convergent validity examination via a multi-trait multi-method approach (Campbell and Fiske, 1959) between the measures was outside of the scope of the current study, we note that the on-the-job embeddedness subscale had a stronger correlation with job satisfaction (*r* = 0.58) than the expected global job embeddedness score (*r* = 0.55). While the strength of the job satisfaction and on-the-job embeddedness relationship was comparable to that reported by Clinton et al. (2012; *r* = 0.61), we expected the relationships between two reliable, theoretically convergent measures to demonstrate a stronger correlation compared to a related, but not theoretically convergent, variable. This finding was, therefore, curious. It may in part explain why the on-the-job embeddedness subscale was unable to account for unique variance in turnover intention, given the shared variance with job satisfaction. While Clinton et al. (2012) previously demonstrated discriminant validity with on-the-job embeddedness and job satisfaction via structural equation modeling, our finding hints at the prospect of notably overlapping variance with an affective determinant of turnover intention. As Mitchell and Lee (2001) posit embeddedness as a non-affective determinant of turnover and turnover intentions, this finding raises further questions about the content captured by Clinton et al.’s (2012) measure. Further research is therefore warranted.

### Stress, Burnout, and Satisfaction at Universities

Descriptive data collected on the known predictors of turnover reflected a discontented and pressured picture of this sector. The sampled Australian university employees scored, on average, in the ‘burnout’ range on the BMS (Malach-Pines, 2005). While previous research has suggested that academic staff report elevated levels of burnout compared to professional staff (Winefield and Jarrett, 2001; Winefield et al., 2003), in this study there was a comparable level of burnout between roles (see **Table 1**). The significant variation in the forms of stress (role ambiguity and conflict) between academic and professional roles reflected findings consistent with that of past research. For example, Winefield et al. (2003) noted that academic positions are relatively more ‘exposed’ compared to other types of work (including professional positions), such that performance, promotion, and recognition are fuelled by externally controlled criteria such as student ratings of teaching efficacy. With funding pattern changes, academic staff members in universities are tasked with seeking external sources of research funding and maintaining a level of research activity in addition to their teaching roles in most instances (Winefield et al., 2003). These conflicting demands may have contributed to the disparity in role conflict scores between academic and professional staff. Due to the bureaucratic structure of Australian universities, ambiguities regarding the manner in which professional staff fulfill tasks may be a source of greater role ambiguity in comparison to academic staff. Our findings, therefore, suggested that the sources of stress for academic and professional staff may vary, indicating a ‘one size fits all’ approach to retention management may be inappropriate for organizational practitioners.

Job satisfaction for the Australian university employees in our sample was lower in comparison to the validation sample reported by Russell et al. (2004). Our findings supported previous findings that suggested below-average job satisfaction scores for university employees (Pop-Vasileva et al., 2011). In contrast to Winefield and Jarrett (2001) and Winefield et al.’s (2003) findings, non-significant differences in job satisfaction between academic and professional staff were observed in our sample. The relationship between burnout, which demonstrated a pattern of elevated scoring in our sample and job satisfaction may have limited the disparity in job satisfaction between university roles in our sample (Hogan et al., 2002). Due to the higher ratings of burnout and lower ratings of job satisfaction noted in our sample of Australian university employees, these areas appear to be prominent potential targets for intervention strategies aimed at mitigating turnover risk for universities.

### Study Limitations

The current study was intended as a preliminary investigation of the predictive utility of job embeddedness in the context of predicting turnover intentions within an Australian university context. Consequently our sample size, while sufficient to address the regression-based analyses conducted in our study, was not sufficient to examine proposed model fit via structural equation modeling. While common method variance is a threat to cross-sectional quantitative research, we have employed several approaches to mitigating the influence of this form of error variance on our findings (see “Design” section).

### Theoretical Implications

The main theoretical implication from our study is that job embeddedness, while a significant determinant of turnover intentions, does not replace ‘established’ determinants of this outcome. In both model variants tested, job satisfaction, burnout, and the perceived availability of job alternatives all accounted for significant variance in turnover intention even after the introduction of the job embeddedness predictors. This finding speaks to the importance of Russell’s (2013) caution to examine incremental variance when considering new predictors of turnover. While transitioning to a construct such as job embeddedness may be in keeping with the contemporary directions of turnover research, researchers should be mindful of existing, valid predictors whose variance job embeddedness does not usurp.

A further theoretical implication arising from our study findings relates to the measurement of embeddedness. Crossley et al.’s (2011) global job embeddedness measure, despite the limitation of being unable to disentangle the on- and off-the-job facets, demonstrated a much stronger case for criterion and incremental validity in predicting turnover intention. The 11% increase in unique explained variance attributed to this determinant in the professional subsample was impressive. Clinton et al.’s (2012) measure was a substantially weaker predictor. Scrutiny of the current items suggests that further development work is required to ensure they measure job embeddedness. For example, one indicator of off-the-job embeddedness “I would be very sad to leave the general community where I am based right now” (Clinton et al., 2012, p. 114) seems to reflect an affective facet, which appears counter to the intent of an embeddedness measure. The on-the-job embeddedness item “I would miss the excitement that this job brings if I left” (Clinton et al., 2012, p. 114), which demonstrated the weakest loading within a sample of IT professionals in Clinton et al.’s (2012) validation article, is another potential choice for revision. As jobs outside of the majority-military context used by Clinton et al. (2012) during their scale development may not be as overtly exciting for employees, this item may also be a target for revision to improve the generalisability of the scale. In summary, the measurement approach provided by Crossley et al.’s (2011) global measure of job embeddedness appeared to be the most favorable reflective measure option for future research, although whether this result holds across different occupational domains would be a valuable area of future research inquiry. Comparing the measurement approaches to embeddedness remains an important avenue in embeddedness research (Zhang et al., 2012).

### Practical Implications

Job embeddedness should be included in future intervention strategies targeting employee retention within universities. Job embeddedness accounts for unique variance in turnover intentions beyond that of other established indicators that organizational practitioners may typically examine (e.g., job satisfaction). This provides an alternative mechanism to consider when designing evidence-based intervention or recruitment strategies for employees. For example, as the perceptions of fit between what the job offers and what the individual wants is a contributor to the ‘glue’ factor of embeddedness, descriptive and transparent outlines of job tasks, responsibilities, and rewards during the recruitment process may make these facets salient to the participant, thereby enhancing embeddedness at onset. Encouraging involvement in social and professional clubs, such as collaborative research groups for academics, may address the links facet of embeddedness. Program evaluation following intervention strategies that try to enhance these embeddedness facets within the workplace, such as measuring embeddedness via Crossley et al. (2011) instrument, could then be conducted to assess change. While these suggestions can be applied in a university context, it is highly probable that they may be tailored to other organizational contexts. Job embeddedness has a complementary place among other traditional targets for intervention such as job satisfaction, and organizational commitment.

## Author Contributions

All authors listed, have made substantial, direct and intellectual contribution to the work, and approved it for publication.

## Conflict of Interest Statement

The authors declare that the research was conducted in the absence of any commercial or financial relationships that could be construed as a potential conflict of interest.

## Footnotes

1 Due to a clerical error the two ‘likely’ items had scale points between 1 and 7. To make these items consistent with Jaros’ (1997) original scale endpoints their values were multiplied by five-sevenths. The revised total score was convergent (*r* = 0.999, *p* < 0.001) with the turnover intention total score created from the original scale values, demonstrated better reliability (α = 0.872 vs. α = 0.852), and was consistent with Jaros’ original scoring pattern. The revised scale was retained for use in the forthcoming analyses.
